# Identification of predilection sites for wild boar carcass search based on spatial analysis of Latvian ASF surveillance data

**DOI:** 10.1038/s41598-023-50477-7

**Published:** 2024-01-03

**Authors:** Lisa Rogoll, Katja Schulz, Christoph Staubach, Edvīns Oļševskis, Mārtiņš Seržants, Kristīne Lamberga, Franz Josef Conraths, Carola Sauter-Louis

**Affiliations:** 1 Institute of Epidemiology, Friedrich-Loeffler-Institut, Federal Research Institute for Animal Health, Südufer 10, 17493 Greifswald-Insel Riems, Germany; 2 Food and Veterinary Service, Peldu 30, Riga, 1050 Latvia; 3 Institute of Food Safety, Animal Health and Environment-“BIOR”, Lejupes 3, Riga, 1076 Latvia

**Keywords:** Risk factors, Viral infection, Epidemiology

## Abstract

Targeted search for wild boar carcasses is essential for successful control of African swine fever (ASF) in wild boar populations. To examine whether landscape conditions influence the probability of finding ASF-positive carcasses, this study analyzed Global Positioning System (GPS) coordinates of Latvian wild boar carcasses and hunted wild boar, extracted from the CSF/ASF wild boar surveillance database of the European Union, and random coordinates in Latvia. Geographic information system (GIS) software was used to determine the landscape type and landscape composition of carcass detection sites and to measure distances from the carcasses to nearest waterbodies, forest edges, roads and settlements. The results of the automated measurements were validated by manually analyzing a smaller sample. Wild boar carcasses were found predominantly in forested areas and closer to waterbodies and forest edges than random GPS coordinates in Latvia. Carcasses of ASF-infected wild boar were found more frequently in transitional zones between forest and woodland shrub, and at greater distances from roads and settlements compared to ASF-negative carcasses and random points. This leads to the hypothesis, that ASF-infected animals seek shelter in quiet areas further away from human disturbance. A detailed collection of information on the environment surrounding carcass detection sites is needed to characterize predilection sites more accurately.

## Introduction

African swine fever (ASF) is a viral disease that constitutes a threat for domestic pigs and wild boar worldwide. The disease is characterised by haemorrhagic fever, which leads to case/fatality ratios of up to 100%^1^.

Since the introduction of ASF virus of genotype II into Georgia in 2007, the disease constantly spread over Europe and Asia, posing a constant threat to wild boar populations and domestic pigs^2,3^. In 2014, the first cases of ASF were detected in Latvia in wild boar in the eastern part of the country close to the border with Belarus^4^. Shortly after, a long-distance jump of the virus to the northern regions occurred, which was most likely human-mediated^5^. In the following years, ASF subsequently spread westwards throughout the country and reached the central part of Latvia in summer 2016^5^. By the end of 2019, the infection was present in wild boar in around 85% of the area of Latvia^6^.

The lasting presence of ASF in wild boar populations increases the risk of introduction of the virus into domestic pig farms^7–10^, which can lead to great suffering in affected pigs and massive socio-economic losses in the pig industry, caused in particular by trade and movement restrictions^11,12^. It is therefore of utmost importance to control the spread of the disease in wild boar populations.

In Latvia, long-lasting infection cycles in wild boar populations with endemic character established^13^. This infection cycle has been described as the “wild boar-habitat cycle” with direct transmission amongst infected and susceptible wild boar as well as indirect transmission through carcasses of infected wild boar present in the habitat^14^. Direct contact of wild boar with the carcasses such as sniffing and poking on carcasses^15^ and forms of cannibalism like consumption of muscles and organs^16^ have been observed. In addition, carcasses may remain infectious over long periods especially at low temperatures^17^ causing local virus persistence in wild boar habitats. For these reasons, rapid search and disposal of wild boar carcasses is considered as one of the most important measures to control ASF in wild boar populations^18^.

Several studies have also shown that the probability of detecting ASF-positive wild boar is clearly higher among wild boar found dead compared to hunted animals^2,19,20^. This finding underlines the importance of passive surveillance through intensive carcass search and sampling.

Nevertheless, the search for wild boar carcasses is a time-consuming, cost-intensive and thus unpopular measure among hunters^21^. Although experts consider carcass search as an effective measure for ASF control, it is rated as less practical^22^. Accordingly, it seems reasonable to develop strategies for conducting carcass search in a more targeted and thus resource-efficient manner. One possible starting point for this is the hypothesis that ASF-infected wild boar prefer different habitats compared to their healthy conspecifics due to symptoms such as fever and depression.

Several studies already tested the hypothesis whether certain characteristics of the habitat increase the probability of finding an ASF-positive carcass and whether this can be used to identify possible predilection sites for carcass search—with variable results. It has been observed that ASF is more likely to occur in forest areas^23–25^. It has been demonstrated that ASF-positive carcasses are more likely to be found in younger forest stands up to 40 years of age in quiet places more distant from roads and forest edges^23^, in areas of transition between woodland and shrub consisting of younger plants^26^ and in cool and moist habitats further away from rivers^26^. Lim et al. reported that the numbers of ASF-infected carcasses were higher in regions with a low heat load index^27^. While it was observed in some studies that indicators of human activity such as the numbers of roads/settlements or human population density were positively associated with the notification of ASF in wild boar^24,27^, others found an inverse influence of human activity on ASF case probability^23,25^.

Based on these variable findings, our study aimed to make use of Latvian surveillance data to identify possible predilection sites for the search of ASF-positive carcasses in Latvia and thus to support the detection of wild boar carcasses.

## Materials and methods

### Data and study area

**Figure 1:**
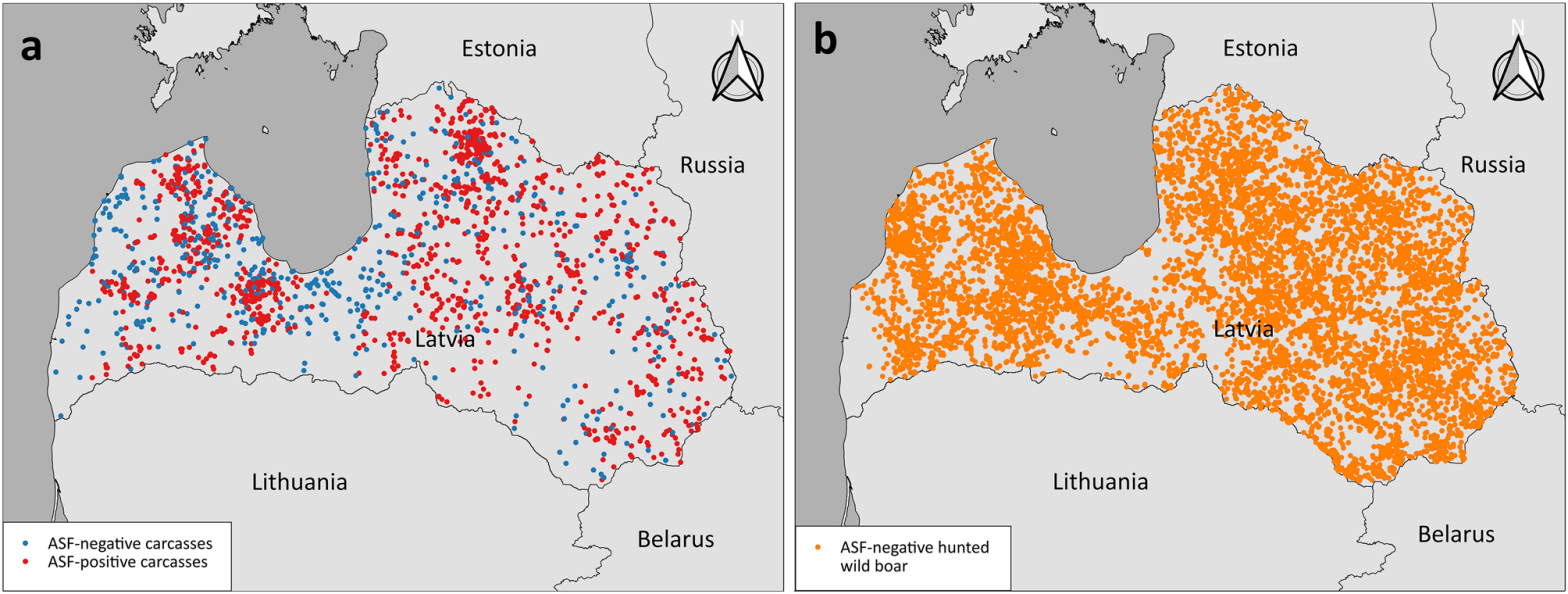
Overview of study area (Latvia) and GPS locations extracted from the EURL CSF/ASF wild boar surveillance database^28^ for (**a**) ASF-negative (n = 606) and ASF-positive (n = 1444) carcasses found dead and (**b**) randomly chosen ASF-negative animals shot dead apparently healthy (n = 9527) from 2014 through to 2021. The map was created using QGIS Desktop 3.20.2^31^.

The data examined in this study originated from the EURL CSF/ASF wild boar surveillance database^28^. The following information was extracted from the database for each record: a unique identifier, the date of finding/shooting of each wild boar, the carcass type (found dead or shot dead), age (as estimated by the reporting person), sex and the results of virological and serological examination (ASF-positive or ASF-negative) as well as Global Positioning System (GPS) coordinates of the place, where the carcass had been detected. In case more than one animal was found/shot in a position with exactly the same coordinates on the same date, the coordinates were only considered once for the analysis. The final data set consisted of 11,577 records including 1444 ASF-positive and 606 ASF-negative wild boar found dead as well as a sample of 9527 ASF-negative wild boar hunted apparently healthy (randomly selected from all records of ASF-negative wild boar hunted apparently healthy in the database) in Latvia from June 2014 through to February 2021 (Fig. 1).

The study area covered the total area of Latvia with a size of 64,589 km^2^^29^ (Fig. 1). According to Corine Land Cover (CLC) 2018 map data (100 m/25 ha resolution)^30^, the majority of the country is covered by agricultural area (39.6%), forests (37.6%) and transitional-woodland-shrub (16.0%). The forest area can furthermore be divided into broad-leaved (8.1%), coniferous (12.8%) and mixed forest (16.7%). The remaining area of Latvia is composed of waterbodies (2.0%), wetlands (2.5%), open spaces (0.1%), urban area (2.0%) and scrub and/or herbaceous vegetation association (0.1%). Based on hunting management units (HMU) of the State Forest Service in Latvia, the majority of agricultural area is composed of cereal fields (0.0% up to 48.7% per HMU), grassland (0.0% up to 43.1% per HMU) and rapeseed fields (0.0% up to 16.9% per HMU) (https://www.silava.lv/images/Petijumi/2022-LVM-Rekomendacijas-briezu-dzimtas-parnadzu-medibu-parvaldibas-pilnveidosanai/2023-LVM-Rekomendacijas-briezu-dzimtas-parnadzu-medibu-parvaldibas-pilnveidosanai-II-etaps.pdf, accessed 1 November 2023).

**Figure 2:**
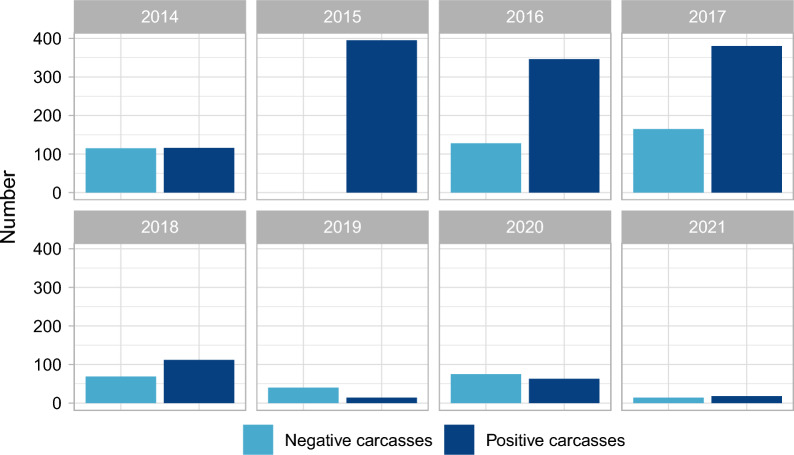
Numbers of records per year (from 2014 to 2021) extracted from the EURL CSF/ASF wild boar surveillance database^29^ for ASF-negative carcasses (n = 606) and ASF-positive carcasses (n = 1444) found dead in Latvia used in the analysis.

In 2015, no ASF-negative carcasses found dead were recorded with unique GPS-locations (Fig. 2). ASF-positive cases mainly originated from the years 2015 to 2017 (Fig. 2). The analysis of the monthly distribution of records (Supplementary Fig. S1) showed that animals were more frequently hunted in winter (n = 5702) than in summer months (n = 3825). In contrast, a higher proportion of carcasses were found in summer months (317 ASF-negative and 826 ASF-positive carcasses) than in winter months (289 ASF-negative and 618 ASF-positive carcasses).

The final data set contained information about the estimated age of the animals shot or found dead in 11,212 of 11,577 cases. The majority of animals (6107 out of 11,212; 55%) was approximately 1 to 2 years old at the time of death (Supplementary Fig. S2). The data set contained information about the sex of the animals for 10,609 of 11,577 cases, out of which 53% were male and 47% female (Supplementary Fig. S3).

In addition, a set of 10,000 random and independent GPS coordinates in Latvia was created as a control, whereby areas of water bodies were excluded.

### Landscape type and landscape composition

The landscape type at the location, where a wild boar carcass had been detected or wild boar were hunted, and the landscape composition in a buffer zone with a radius of 3 km around the location to reflect potential moving distances and home ranges of wild boar^32,33^ were analyzed using the geographic information system (GIS) software ArcGIS ArcMap 10.8.1^34^. The CLC map data was used within the projected coordinate system LKS92/Latvia TM (EPSG:3059). For both analyses, existing CLC categories were grouped together to the following landscape categories: (i) Forest (for analysis of landscape composition divided into broad-leaved, coniferous and mixed forest), (ii) agricultural area (including fields, arable land, crops and pastures), (iii) transitional woodland-shrub, (iv) waterbodies, (v) wetlands, (vi) open spaces with little or no vegetation (e.g. beaches, dunes, rocks), (vii) urban areas and (viii) scrub and/or herbaceous vegetation associations (including moors and heathland, natural grassland and sclerophyllous vegetation). For the analysis of the landscape type of detection sites and random points, a landscape category was allocated to each location using the ArcMap tool “Spatial Join”. To analyze the landscape composition around the locations, the proportion of the area of different landscape categories in the buffer zones was calculated using the ArcMap tool “Intersect” and the field calculator.

Since CLC only provides landcover information for European countries, the landscape composition of buffers lying close to the border with Belarus and Russia could not be analyzed completely due to lack of data. This affected 115 records from the database and 87 random points. The respective records were excluded from the evaluation of the landscape composition.

### Distance measurements

In a second step, the shortest distances of the locations, where wild boar carcasses had been detected or wild boar were hunted, to the next waterbody, road, settlement and forest edge were measured automatically using the GIS software QGIS Desktop 3.20.2^31^ and the plug-in “NNJOIN”^35^. For polygon layers, the shortest distance to the external boundary of the polygon was measured for both locations within and outside the polygon.

For the distance to the next waterbody, map data for rivers as well as inland and marine waterbodies from a Latvian topographic map (25 m resolution)^36^ were used. To measure the distance to the next road, we used OpenStreetMap (0.4 m resolution)^37^. Only major roads suitable for cars were included in the measurements (feature classes “trunk”, “primary”, “secondary”, “tertiary” as well as roads named with a “V”). Data for measuring the distance to the next settlement was obtained from the Copernicus European Settlement map (2 m resolution)^38^. The distance to the next forest edge was measured by using the Copernicus Forest Type map (10 m resolution)^39^. Thereby, the forest edge corresponded to the interface between forest and other habitat types.

### Manual analysis

Manual analyses were performed to validate the results of the automated analyses of landscape types and distance measurements. Therefore, a smaller sample (n = 599) of ASF-positive carcasses (n = 249), ASF-negative carcasses (n = 175) and ASF-negative hunted wild boar (n = 175) was randomly selected from the original data set. For these locations, landscape type was examined and distances were measured manually within the record viewer of the EU CSF/ASF wild boar surveillance database using integrated orthophotos and a ruler tool. Beforehand, criteria for landscape analysis were set (Supplementary Table S1 and Supplementary Table S2).

### Statistical analysis

Differences between ASF-positive carcasses, ASF-negative carcasses, ASF-negative hunted wild boar and random points were analyzed using non-parametric test methods, since the data were not normally distributed (tested with Kolmogorov–Smirnov-Test). Fisher’s Exact and Kruskal–Wallis tests with subsequent pairwise Mann–Whitney-U-Test with Bonferroni correction were used for group comparisons. P-values of less than 0.05 were considered statistically significant.

Based on the date of detection or shooting of wild boar, respectively, two subgroups of the final data set were formed and tested for seasonal differences: (a) entries from summer months (April to September) and (b) entries from winter months (October to March). These analyses of the landscape type and landscape composition as well as distance measurements were performed with the whole data set and separately for the summer- and winter group.

In order to account for interactions between variables, multivariable logistic regression was performed to identify significant predictors, which increase the chance of finding ASF-positive carcasses. The outcome variable was the infection status of a carcass found dead (ASF-negative vs. ASF-positive). The tested predictors were the distances to certain landscape features (scaled to 100 m steps) and proportions of different landscape types (scaled to 10% steps).

To test for spatial autocorrelation, Moran’s I, Geary’s C and semi-variograms were calculated on the standardized deviance residuals of ordinary univariable generalized mixed models (GLM), as described by Cressie^40^ and Diggle and Ribeiro^41^. To correct for spatial autocorrelation univariable generalized estimated equation (GEE) models were implemented^42^. The predictive quality of the univariable models was evaluated by calculating the area under the curve (AUC) of receiver operating characteristic (ROC) plots. Only predictors with p-values below 0.2 or AUC above 0.55 were retained in the multivariable GEE model. The final model was developed using backward elimination, whereby only predictors with p-values below 0.05 were included in the final model.

All statistical evaluations were conducted with the statistic software R^43^ using Rstudio 4.0.3^44^ as an interface. The packages tidyverse^45^, dplyr^46^ and lubridate^47^ were used for data management and the package ggplot2^48^ was used for visualizing of results. The package geoR was used to calculate semivariograms^49^, the packages gee^50^ and MASS^51^ were used to implement GEE models, and the package epiDisplay^52^ was used to create ROC plots. Supplementary Table S3 provides an overview of datasets and statistical methods used for each step of the analyses.

## Results

### Landscape type

**Figure 3:**
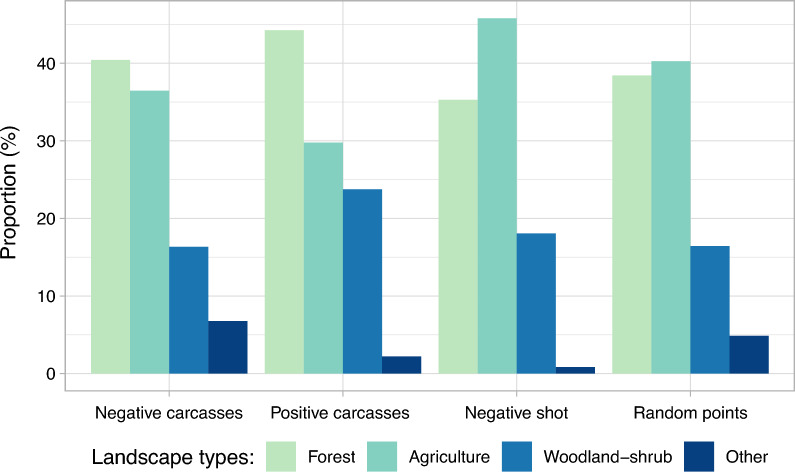
Proportions of landscape types (forest, agricultural area, transitional woodland-shrub and other locations) for ASF-negative carcasses (n = 606), ASF-positive carcasses (n = 1444), ASF-negative wild boar shot apparently healthy (n = 9527) between 2014 and 2021 and random points (n = 10,000) in Latvia. Other locations include waterbodies, wetlands, open spaces with little or no vegetation (e.g. beaches, dunes, rocks), urban areas and scrub and/or herbaceous vegetation associations (including moors and heathland, natural grassland and sclerophyllous vegetation).

The distribution of different landscape categories (Fig. 3 and Supplementary Table S4) of detection sites of ASF-positive carcasses differed statistically significantly from those of ASF-negative carcasses (p < 0.001). Although carcasses were often found in the forest, the proportion was higher for ASF-positive carcasses than for ASF-negative carcasses. By contrast, a higher proportion of ASF-negative carcasses was found on fields. The number of animals found in transitional woodland-shrub was higher for ASF-positive carcasses than for ASF-negative carcasses. In contrast to that, animals were mainly shot on fields and less frequently in forests and transition zones. In addition, the distribution of the landscape categories of random points differed statistically significantly from the results of ASF-positive carcasses found dead and ASF-negative hunted wild boar (both p < 0.001). Results of all pairwise comparisons are shown in Supplementary Table S5.

Only small proportions of records (animals and random points) were associated with other landscape types, such as urban areas, waterbodies, wetlands, open spaces with little or no vegetation and scrub and/or herbaceous vegetation associations (Supplementary Table S4). The proportion of ASF-positive carcasses found in urban areas (0.6%) was smaller compared to negative carcasses (3%) and random points (2%).

The analysis of the two seasonal subgroups showed that the distribution of detection or hunting sites, respectively, differed statistically significantly in the Fisher’s Exact test between summer and winter months for ASF-positive carcasses (p = 0.003) and ASF-negative hunted wild boar (p < 0.001) (Supplementary Table S6).

### Landscape composition

**Figure 4:**
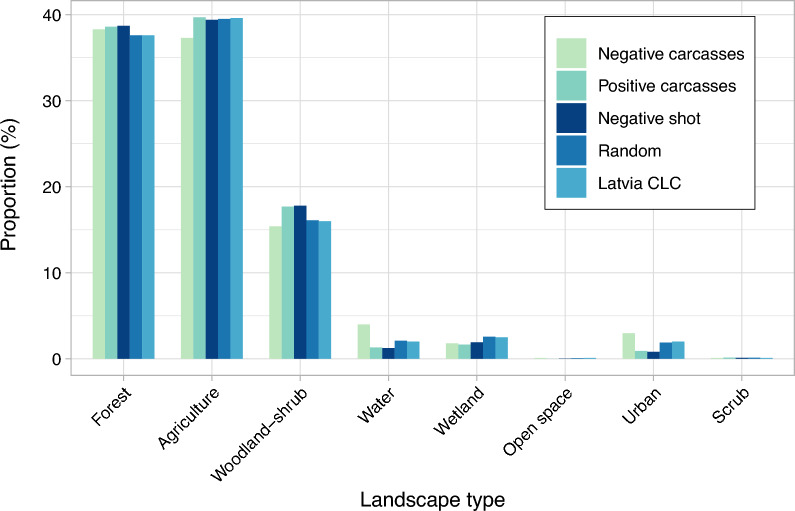
Mean proportions (in %) of different landscape types in a buffer zone with a radius of r = 3 km around the locations for ASF-positive carcasses (n = 1423), ASF-negative carcasses (n = 605), ASF-negative animals shot apparently healthy (n = 9434) from 2014 to 2021, random points (n = 9913) and Latvian landscape.

The main landscape components in a buffer zone of 3 km radius around the locations of ASF-positive (n = 1423) and -negative carcasses (n = 605), ASF-negative hunted animals (n = 9434) and random points (n = 9913) were forests, agricultural area and transitional woodland-shrub for all study groups (Fig. 4). The proportions of water, wetland, open spaces, urban areas as well as scrub and/or herbaceous vegetation were rather small in the buffer zones according to the CLC data.

The proportion of total forest area in the buffer zones was similar for ASF-positive carcasses, ASF-negative carcasses and hunted wild boar (p = 1.000 for all pairwise tests), whereas differences were observed in the proportions of different forest types: Around ASF-positive carcasses, the proportion of coniferous forest was significantly smaller (p = 0.001) and the proportion of broad-leaved (p < 0.001) and mixed forest (p = 0.003) significantly larger compared to ASF-negative carcasses. ASF-positive carcasses (p = 0.02) and ASF-negative hunted wild boar (p < 0.001) had a significantly larger median proportion of forest area in their buffer zones than the random points.

The proportion of agricultural area was significantly smaller for ASF-negative carcasses than for ASF-negative animals shot dead (p = 0.07). All remaining comparisons failed to result in statistically significant differences regarding the proportions of agricultural area (p > 0.05 for all pairwise tests regarding ASF-negative carcasses, ASF-positive carcasses, ASF-negative hunted animals and random points).

ASF-positive carcasses had a significantly greater proportion of transitional woodland shrub in their 3 km-buffer-zone than ASF-negative carcasses (p < 0.001), whereas the random points had a significantly smaller median proportion of transitional woodland-shrub than ASF-positive carcasses (p < 0.001) and ASF-negative hunted animals (p < 0.001).

In contrast, the proportion of urban area was significantly smaller for ASF-positive carcasses (p < 0.001) and hunted wild boar (p < 0.001) than for ASF-negative carcasses. The random points had a statistically significantly greater proportion of urban areas in their buffer zones than ASF-positive carcasses (p < 0.001) and hunted ASF-negative animals (p < 0.001), but a significantly smaller proportion of urban areas than ASF-negative carcasses (p < 0.001).

The analysis of seasonal differences in the landscape composition can be found in Supplementary Table S7.

### Distance measurements

The results of the distance measurements of the locations of the carcasses, the hunted wild boar and the random points are displayed Table 1.

**Table 1:** Median distances (in m) of ASF-negative carcasses, ASF-positive carcasses, ASF-negative wild boar shot dead and random points to the next waterbody, road, settlement and forest edge in Latvia from 2014 through to 2021.

	ASF-negative carcasses	ASF-positive carcasses	ASF-negative hunted	Random points
	(n = 606)	(n = 1444)	(n = 9527)	(n = 10,000)
Waterbody	116	121	120	129
Road	611	1179	1224	1054
Settlement	356	453	497	462
Forest edge	53	49	51	75

Animals of all three groups were found or shot, respectively, statistically significantly closer to forest edges than the random points (p < 0.001 for all pairwise tests). No significant differences were detected between ASF-negative carcasses, ASF-positive carcasses and ASF-negative hunted wild boar (p > 0.05 for all pairwise tests). Out of all animals, 70% were found or shot within a distance of 100 m to the nearest forest. Among the carcasses found dead, less than 5% were found in distances over 300 m to the next forest edge.

Wild boar were shot statistically significantly closer to waterbodies than the random points (p < 0.001). No statistically significant difference between ASF-positive and ASF-negative carcasses was detected regarding the distance to waterbodies (p = 1.000). The majority of analyzed locations (67%) was within a 200 m distance to the next waterbody, although the proportion was higher for ASF-positive carcasses (68%) and ASF-negative carcasses (70%) compared to the random points (65%). Only 6% of ASF-positive and 4% of ASF-negative carcasses were found beyond a distance of 500 m to the nearest waterbody.

Furthermore, ASF-positive carcasses and hunted animals were found statistically significantly further away from roads and settlements than ASF-negative carcasses (p < 0.001 for all pairwise tests). The greatest differences occurred in the distance to roads: 23% of ASF-positive and 46% of ASF-negative carcasses were found within a distance of 500 m to the next road. The random points were statistically significantly closer to roads than hunted wild boar (p < 0.001), but further away than ASF-negative carcasses (p < 0.001). Regarding the distance to settlements, the random points were statistically significantly closer than animals shot dead (p = 0.002), but further away than ASF-negative carcasses found dead (p < 0.001). The distance of ASF-positive carcasses and random points to settlements and roads was not statistically significantly different (p > 0.05).

In addition, the distances of ASF-positive carcasses and animals shot dead to waterbodies, roads, settlements and forest edges were not significantly different (p > 0.05 for all pairwise tests).

Regarding seasonal differences, ASF-negative carcasses were found significantly closer to roads (p < 0.001) in winter months (402 m) than in summer months (816 m) (Supplementary Table S8).

### Generalized estimation equation model

Ordinary univariable GLM models showed spatial correlation, which was tested by using Moran’s I, Geary’s C and semi-variograms calculated on the standardized deviance residuals (Supplementary Tables S9 and S10).

**Figure 5:**
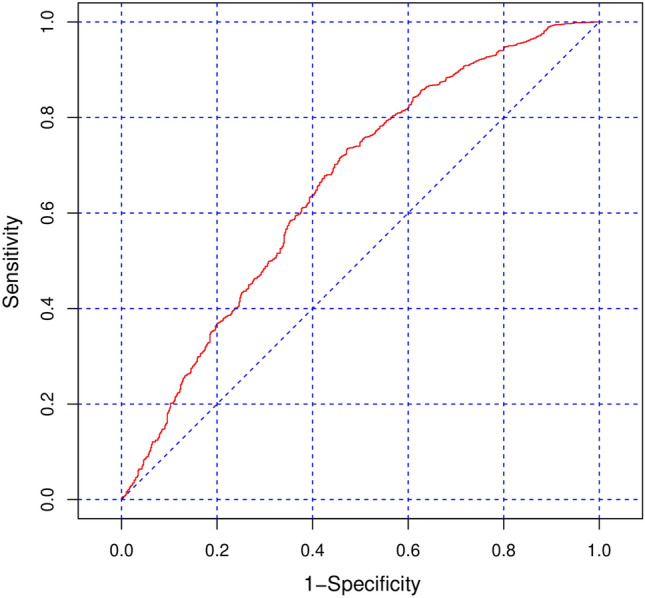
ROC plot of the final multivariable GEE model that included the predictors distance to forest edge, distance to roads, proportion of open space, proportion of waterbodies and proportion of broad-leaved forest. AUC = 0.6575.

Univariable GEE models (Supplementary Table S11) examined potential factors that were associated with the infection status of the carcasses that were found dead (ASF-negative vs. ASF-positive). Based on the selection criteria of AUC and significance, the distance to waterbodies, the proportion of total forest area, agricultural area, scrub and mixed forests were excluded from the model. The proportion of wetlands was also excluded, since the AUC of the model was below 0.5. The final multivariable model (Table 2) with an AUC of 0.6575(Fig. 5) showed that the increasing distance to road had a positive effect on the chance of finding an ASF-positive carcass, whereas increasing distance to the forest edge, the proportion of open space and waterbodies and mixed forests had a negative effect. The semi-variogram of the GLM standardized deviance residuals used to correct the final GEE model, showed that the practical range^40^ of the spatial correlation was 27.48 km (Supplementary Fig. S4).

**Table 2:** Results of the multivariable GEE model showing the estimates, p-values, odds ratios (OR) and approximate 95% confidence intervals (CI) of predictors based on naïve standard errors.

Predictor	Estimate	P-value	OR (95% CI)
Intercept	0.4749	0.039	
Distance to road	0.0394	< 0.001	1.0400 (1.0330, 1.0480)
Distance to forest edge	− 0.0469	0.018	0.9542 (0.9180, 0.9919)
Proportion of open space	− 9.1972	0.030	0.0001 (2.547 * 10^–8^, 0.4032)
Proportion of waterbodies	− 0.3238	< 0.001	0.7234 (0.6082, 0,8604)
Proportion of mixed forest	− 0.0862	0.018	0.9174 (0.8541, 0.9854)

The outcome variable was the infection status of the carcasses found dead (ASF-negative [n = 605] versus ASF-positive [n = 1423]) in Latvia from June 2014 through to February 2021.

### Manual analysis

The evaluation of the smaller sample regarding the landscape type of carcass detection and hunting sites showed that most results were similar in both methods without statistically significant differences (Supplementary Table S12): ASF-positive carcasses were predominantly found in forests and transitional woodland-shrub areas, while the ASF-negative carcasses were found more frequently in agricultural areas and in other landscape types. Hunted animals were more often shot on agricultural areas than in forests.

There was a considerable difference between the manual evaluation and the automated approach regarding the ASF-positive carcasses found in forests (59% versus 48%) and in transitional woodland-shrub (20% versus 29%). However, the overall percentage of ASF-positive carcasses found in forests and transitional woodland-shrub combined was similar (79% versus 77%) with both methods.

Another difference was observed in the group of ASF-negative carcasses that were found in other landscape categories. This proportion was in the manual measurement twice as high (14%) than in the automated measurement (7%). In the manual measurement, 5% of the ASF-negative carcasses were found in urban areas, 3% in water bodies and 2% each on roads, in wetlands and on beaches. In the automated measurement, 4% of the ASF-negative carcasses were found in urban areas and 1% each in water bodies, wetlands and on beaches. The detection of carcasses found on roads was not possible in the automated evaluation, since the CLC map data does not contain information about infrastructure.

The comparison of manual and automated distance measurements (Table 3) using the Mann–Whitney-U test showed statistically significant differences in the distances to waterbodies (p < 0.001), settlements (p = 0.003) and forest edges (p = 0.002), but not in the distance to roads (p = 0.797). The locations were 145 m (median) closer to waterbodies (1st quartile Q1 = − 458 m, 3rd quartile Q3 = − 3 m), 5 m further away from roads (Q1 = − 1 m, Q3 = 11 m), 3 m closer to settlements (Q1 = − 190 m, Q3 = 34 m) and 3 m further away from forest edges (Q1 = − 6 m, Q3 = 26 m) in the automated measurements compared to the manual measurements.

**Table 3:** Comparison of the median distances (m) to the nearest waterbodies, roads, settlements and forest edges measured manually and in an automated way for a subset of ASF-negative, ASF-positive wild boar carcasses and ASF-negative hunted wild boar in Latvia from 2014 through to 2021.

	ASF-negative carcasses		ASF-positive carcasses		ASF-negative hunted wild boar	
	(n = 175)		(n = 249)		(n = 175)	
	Measurement					
	Manual	Automated	Manual	Automated	Manual	Automated
Waterbody	290	89	347	94	354	110
Road	704	659	1320	1348	1360	1365
Settlement	410	370	573	535	550	430
Forest edge	40	53	41	60	40	46

## Discussion

The continued spread of ASF constitutes a threat for wild boar and domestic pigs worldwide and sets a challenge for veterinary authorities, hunters and farmers. Among different measures to control the spread of ASF in wild boar, one essential strategy is the quick search for and removal of wild boar carcasses, which requires substantial financial and personal resources. We aimed to identify predilection sites for the search of wild boar carcasses to optimize the searches and save resources. To this end, a data set of 2050 GPS locations of detection sites of wild boar carcasses from Latvia, where ASF occurred since 2014, was analyzed to examine whether certain characteristics of the landscape influence the probability of finding a carcass of ASF-infected wild boar. All records of ASF-positive wild boar were considered in the evaluation, regardless of the testing method (PCR or serology). Three records out of 1444 had tested serologically positive, but virologically (i.e. PCR) negative for ASF. It is therefore unclear whether these three animals died of ASF. However, due to the small number, their influence on the overall results is regarded as extremely limited if not negligible.

Moreover, a dataset of 9527 GPS locations of hunting sites was analyzed. Overall, it was assumed that hunting locations represent wild boar habitats, as it seems reasonable that hunters predominantly hunt wild boar in places where wild boar are abundant. However, a bias in the data towards hunters’ choice of where to hunt wild boar must be considered.

Since the landscape structure of Latvia is relatively homogenous and primarily consists of agricultural area and forests, a data set of 10,000 random GPS locations was created and analyzed in the same manner to put the possible results and correlations in relation to the general landscape structure present in Latvia.

When interpreting the study results, the possible accuracy of GPS data and available map data must be considered. It has been reported that GPS devices can reach precisions of under 10 m^53^. However, this precision can vary considerably and may be negatively influenced by lack of satellite availability and dense forest canopy cover^54^. Accordingly, a certain inaccuracy of GPS data submitted to the surveillance database must be tolerated, especially regarding locations in forests. However, this inaccuracy can eventually be neglected when put into relation to the resolution of the CLC map data. The minimum mapping unit of these data is 25 ha (500 × 500 m) and does therefore not capture smaller landscape features. Nevertheless, CLC provides good information on landscape composition with a coverage of 100% within Europe and has been used for similar purposes in several other studies^24,26,55,56^.

Furthermore, inaccuracies have to be considered for the map data used for distance measurements: The median difference between automated and manual measurements of the distance to waterbodies suggested that there were discrepancies between map data and orthophotos. Random checks of GPS locations with large individual differences in this comparison revealed that small waterbodies and waterbodies covered by dense forest were easily overlooked or impossible to see in orthophotos. The median differences in the measurements of the distances to roads, settlements and forest edges were rather small, but the interquartile ranges suggested that outliers with large individual differences occurred. Random checks of GPS locations showed that this was at least in part due to incorrect classification of settlements by the European Settlement Map. Nevertheless, the median differences showed that individual divergences in both directions were eventually balanced, leading to the conclusion that the automated method is suitable for analysing large surveillance data sets.

Overall, the established GEE model (ASF-negative vs. ASF-positive carcasses) identified statistically significant factors influencing the probability of finding an ASF-positive carcass. According to the model, it was less likely to find an ASF-positive carcass with increasing distance to forest edges and increasing proportion of open spaces around the carcass. Combined with the fact that the proportion of carcasses found in forests was higher among ASF-infected wild boar than for non-infected, this might lead to the hypothesis that infected animals search for shelter in forest areas. This is in accordance with the results of other studies that observed associations between occurrence of ASF and forest coverage^24,25,55^. However, forest areas with nut-bearing trees and thickets generally represent a preferred natural habitat for wild boar, as they provide protection from predators and various food sources^57–59^. In our study, all carcasses were found close to forest edges, irrespective of their infection status. This was also observed by Cukor et al., who found that the vast majority of carcasses was found in forests and within a distance of up to 200 m to forest edges^23^. In both studies, that of Cukor et al. and in our own investigations, only few carcasses were found beyond distances of 500 m to the forest edge, indicating that forest edges are a potential key area for the detection of ASF-positive carcasses. Yet, these results may be biased by the fact that the search for carcasses is related to the accessibility of the terrain. Since peripheral areas of a forest may be easier to access, it could be more likely to find wild boar carcasses in these areas. This may also be a reason why increasing proportions of mixed forest area decreased the chance of finding a positive carcass in our model, since the density of trees and understorey vegetation are usually higher deep inside the forest and therefore limit detectability.

ASF-positive carcasses were found more frequently in areas of transitional woodland-shrub than negative carcasses, although the predictor was not statistically significant in the final model. They also had a greater proportion of this landscape type in their buffer zones compared to the random points. Similarly, Allepuz et al. identified an increased likelihood of finding positive carcasses in areas of transition between woodland and shrub^55^. These results may suggest that infected wild boar prefer to stay in border regions of forests to seek for shelter.

Beyond that, some studies concluded that ASF-infected animals might preferably stay close to water sources to cool down their body temperature if they have fever, which is a common clinical sign in ASF-infected pigs^23,24,26^. Yet, Allepuz et al. did not identify the distance to waterbodies as a statistically significant factor for the probability of finding ASF-positive carcasses^55^. Our model predicted a decreasing probability of finding an ASF-positive carcass with increasing proportion of waterbodies in the buffer zone around the location, which is most likely due to an artefact, since increasing proportions of waterbodies decrease the area that can be searched for carcasses due to the water coverage. Therefore, the probability to find a positive carcass or a carcass may generally decrease with increasing proportion of waterbodies. In our study, this was particularly obvious for areas close to the coast of the Baltic Sea, which contained large proportions of waterbodies.

Similar to the results of Cukor et al.^23^, we found the majority of carcasses (70%) within a distance up to 200 m to the nearest waterbody, regardless of the infection status of the carcasses. Moreover, 64% of the random points were found within a distance of up to 200 m to the next waterbody, which implicates that Latvia is a water-rich country in general. It has a dense network of lakes, rivers, streams and ditches with a total surface area of approximately 2300 km^2^^29^, which represents 3.6% of the total area of the country. This implies that wild boar behavior and movement in Latvia might not be strongly influenced by the distance to water bodies in general, since it has been proven that wild boar adapt easily to the circumstances of their habitat and that the proximity to water bodies is more relevant for wild boar in dry regions than in water-rich areas^32,59^.

In addition, climate conditions seem to influence the dependence of places, where wild boar chose their death bed, on water sources available nearby^23^. It has also been demonstrated that meteorological conditions such as temperature and precipitation, generally influence the spatial behavior of wild boar^32,58^. Some studies also observed a higher probability of ASF-occurrence in regions with lower mean temperatures^26,27^. Also, in our study, ASF-positive carcasses were found slightly closer to waterbodies in summer as compared to winter months. Yet, the results of the seasonal comparison of carcass finding sites in this study must be interpreted with care, since the analysis is based on the carcass detection dates, which might not necessarily be identical with season at the date of death. Considering the actual climate conditions at the time of death would require to assess the time between the death of a wild boar and its detection, the so-called post-mortem interval, based on the decomposition of the carcass. Such data was not available for the present study. Probst et al. showed that the decomposition process is highly variable and dependent on climatic and landscape conditions^60^, which makes the estimation of post-mortem intervals difficult. Nevertheless, it is known that wild boar spatially adapt themselves to the seasonal variability of available food and shelter^59,61^. During the growing season in summer months, they move closer to fields and agricultural areas to feed on crops, while they dwell in winter especially in forests with broad-leaved and nut-bearing trees that provide food sources^59^. This effect was also visible in our results, since ASF-positive carcasses and hunted animals had a higher proportion of agricultural areas in their environment during summer months.

Besides, our results may also indicate that human activities have an impact on the probability of finding a carcass of an ASF-infected wild boar, based on the analysis of the distance to roads and settlements as well as the proportion of urban areas around finding sites. Negative carcasses were found closer to roads compared to ASF-positive carcasses, especially in winter months. Similarly, Cukor et al. observed that negative carcasses were found significantly closer to roads^23^. This might be due to the fact that road traffic accidents, apart from hunting, are a common cause of death of wild boar^62^, especially in darker winter months. The ASF-negative wild boar found dead might therefore in many cases originate from road traffic accidents and may have been incorrectly classified in the EURL CSF/ASF wild boar surveillance database as a wild boar found dead. It has already been pointed out by Schulz et al. that road traffic accidents are most likely underreported in the database and only few cases have been reported during the whole study period from 2014 to 2021^63^. However, this finding may be biased by the fact that dense snow coverage in winter months might reduce the chances of detecting carcasses far from roads and paths. Nevertheless, the results may also indicate that infected animals are less mobile due to the severity of ASF symptoms and the inclination of infected wild boar to hide as much as possible from human disturbance.

The evaluations showed similarities between detection sites of ASF-positive carcasses and hunting locations of ASF-negative wild boar. Although the hunting sites differed from finding sites of carcasses, since the majority of hunted animals was shot on fields, the other results do not imply huge differences. This may be due to the fact that hunters are often the ones who find and report wild boar carcasses during their hunting activities. Moreover, active surveillance in Latvia included the sampling of hunted wild boar within a radius of 8 to 20 km around newly detected ASF cases^6^, which have led to similarities in landscape composition in the buffer zones of wild boar in these areas.

Although the odds ratios of the predictors in our final logistic regression model were small, we can assume that the calculated p-values are truly significant, since we also corrected the model to account for the spatial correlation^64^. The predictive quality (AUC) of our model was moderate, which may be caused by the influence of many unknown factors, such as different surveillance efforts as well as the temporal course of the spatial spread of ASF during the study period.

In conclusion, we found that forest edges and clearings, as well as bushlands close to forests were predilection sites for the detection of wild boar carcasses in Latvia. Since wild boar can adapt to various habitats and their abundance is always influenced by local circumstances like availability of food resources and level of human interference^65^, the results of this study may not be valid for other study areas with different habitat conditions. However, our results are in many aspects similar to those of other studies on the topic^24–26^ and highlights the consistency of the influence of certain landscape characteristics across different study areas, time periods and methods. It seems therefore possible to use similar data from other regions to define predilection sites, on which the search for wild boar carcasses can focus in ASF-affected areas to save resources.

### Supplementary Information

Supplementary Information.

## Data Availability

The data that support the findings of this study are available from the responsible authority in Latvia upon reasonable request.
