# Supplementary material for: Identification of predilection sites for wild boar carcass search based on spatial analysis of Latvian ASF surveillance data
Source: Sci Rep. 2024 Jan 3;14:382. doi: 10.1038/s41598-023-50477-7 (PMC10764341; doi:10.1038/s41598-023-50477-7)
Supplement: Supplementary file 1 — Supplementary Information. [file 41598_2023_50477_MOESM1_ESM.pdf]

Identification of predilection sites for wild boar carcass search based on spatial analysis of Latvian ASF surveillance data - Supplementary Information

Material and methods

Supplementary Table S1: Criteria defined and applied for landscape categorization of carcass detection or hunting sites of wild boar for the manual measurements.

Landscape type	Definition
Forest	Area densely overgrown with trees.
Agricultural area	Area with open, little overgrown surface.
Forest-field transition	Areas where the landscape was covered with bushes and changed from areas densely overgrown with trees to an open, little overgrown surface.
Other	All other landscape types, e.g. waterbodies, beaches, roads and urban areas.

Supplementary Table S2: Criteria that were defined and applied in manual measurements of distances of carcass detection or hunting sites of wild boar to the next waterbody, road, settlement and forest edge.

Landscape feature	Definition
Waterbody	Marine and inland waterbodies and rivers that could be clearly identified as such and appeared to be a permanent source of water throughout the year. Waterbodies were considered as clearly identifiable if they…<br>1) had a nametag,<br>2) or appeared to be a flowing water with width of at least 3 m and associated/connected with a larger network of waterbodies in the satellite image<br>3) or appeared to be a stagnant water with a width of at least 3 m and a length of at least 10 m in the satellite image.
Road	Tarred highways and main roads, including conjunctive roads that seemed to be an important connection between cities or highways.
Settlement	A small arrangement of at least two buildings that appeared to be inhabited and intact. The distance to the nearest house of the arrangement was measured.
Forest edge	Demarcation line where tree overgrowth ended.

Supplementary Table S3: Overview of material and methods used in the analyses.

**Analysis**	**Aim**	**Data sets**	**Statistical analysis**
Landscape type	Determination of the landscape type at the location of ASF-positive and ASF-negative carcasses, ASF-negative hunted wild boar and random points	1. ASF-positive carcasses (n = 1,444)<br>2. ASF-negative carcasses (n = 606)<br>3. ASF-negative hunted animals (n = 9,527)<br>4. Random points (n = 10,000)	• Group comparisons between ASF positive carcasses, ASF-negative carcasses, ASF-negative hunted wild boar and random points<br>• Fisher's Exact and Kruskal-Wallis tests with subsequent pairwise Mann-Whitney-U-Test with Bonferroni correction
Landscape composition	Calculation of the proportion of different landscape types in an area with a 3 km radius around locations of ASF-positive and ASF-negative carcasses, ASF-negative hunted wild boar and random points	1. ASF-positive carcasses (n = 1,423)*<br>2. ASF-negative carcasses (n = 605)*<br>3. ASF-negative hunted animals (n = 9,343)*<br>4. Random points (n = 9,913)*	
Distance measurements	Measuring the distance of locations of ASF-positive and ASF-negative carcasses, ASF-negative hunted wild boar and random points to the next forest edge, waterbody, road or settlement	1. ASF-positive carcasses (n = 1,444)<br>2. ASF-negative carcasses (n = 606)<br>3. ASF-negative hunted animals (n = 9,527)<br>4. Random points (n = 10,000)	
Seasonal differences	Investigation of seasonal difference in landscape type, landscape composition and distance measurements of locations of ASF-positive carcasses, ASF-negative carcasses and ASF-negative hunted wild boar	Subgroups for summer (April to September) and winter (October to March) of data sets 1, 2 and 3	• Comparisons between summer and winter subgroups<br>• Fisher's Exact and Kruskal-Wallis tests with subsequent pairwise Mann-Whitney-U-Test with Bonferroni correction
Multivariable GEE model	Identification of significant predictors that increase the chance of finding an ASF-positive carcass	1. ASF-positive carcasses (n = 1,423)*<br>2. ASF-negative carcasses (n = 605)*	• Multivariable generalized estimated equation (GEE) model
Manual Measurements	Manual validation of GIS analysis of landscape type and distance measurements	1. ASF-positive carcasses (n = 249)<br>2. ASF-negative carcasses (n = 175)<br>3. ASF-negative hunted animals (n = 175)	• Comparisons between manual and automated analysis<br>• Fisher's Exact and Kruskal-Wallis tests with subsequent pairwise Mann-Whitney-U-Test with Bonferroni correction

** Since CLC only provides landcover information for European countries, the landscape composition of buffer zones lying close to the border with Belarus and Russia could not be analyzed completely due to lack of data. The respective records were excluded from the evaluation of the landscape composition.*

Data description

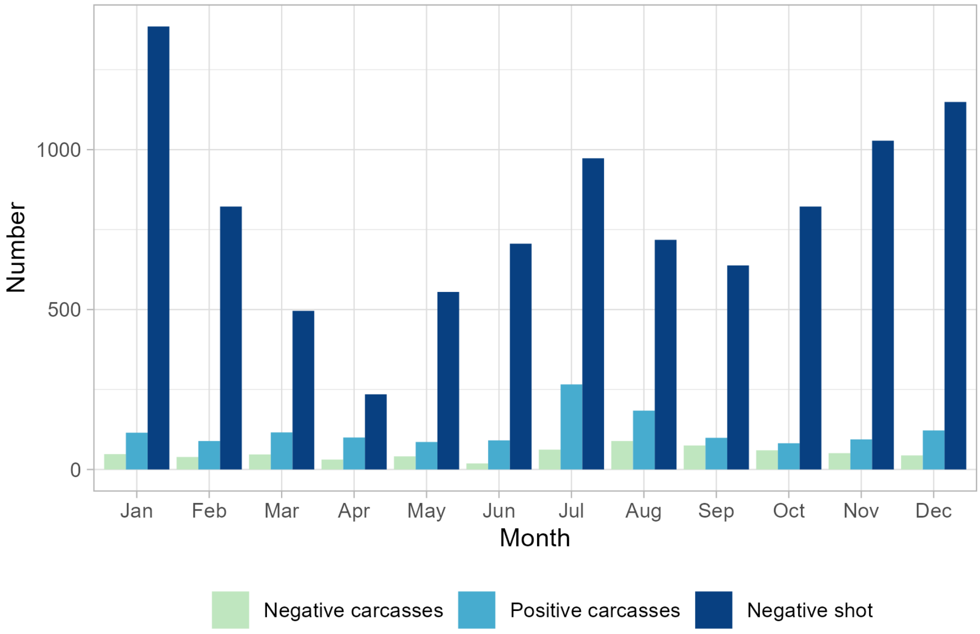

Supplementary Figure S1: Number of records per month (aggregated for all years) according to the date of carcass detection or shooting as entered into the EU database for ASF-negative carcasses (n = 606), ASF-positive carcasses (n = 1,444), ASF-negative hunted wild boar (n = 9,527) throughout the study period from 2014 through to 2021.

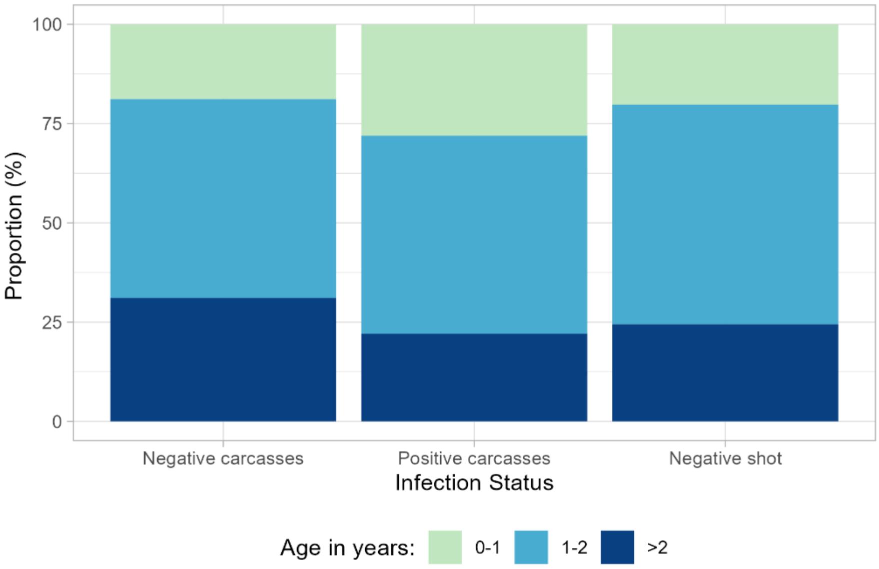

Supplementary Figure S2: Proportion (in %) of different age groups (0 to 1 year, 1 to 2 years, over 2 years old) for ASF-negative carcasses (n = 531), ASF-positive carcasses (n = 1,265) and ASF-negative hunted wild boar (n = 9,416).

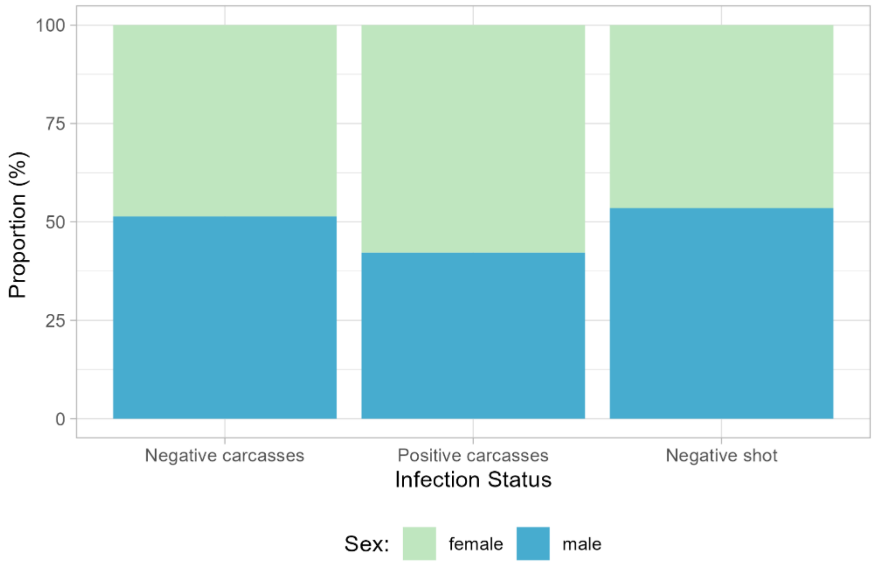

Supplementary Figure S3: *Proportions (in %) of female and male ASF-negative carcasses (n = 403), ASF-positive carcasses (n = 773) and ASF-negative wild boar shot dead (n = 9,433).*

Landscape type

Supplementary Table S4: Proportion of carcasses (in %) that were found or hunted in forests, agricultural area, transitional woodland-shrub or other locations for ASF-negative carcasses, ASF-positive carcasses, ASF-negative hunted wild boar and random points. Other locations include waterbodies, wetlands, open spaces with little or no vegetation (e.g. beaches, dunes, rocks), urban areas and scrub and/or herbaceous vegetation associations (including moors and heathland, natural grassland and sclerophyllous vegetation).

	ASF-negative carcasses (n = 606)	ASF-positive carcasses (n = 1,444)	ASF-negative hunted wild boar (n = 9,527)	Random points (n = 10,000)
Forest	40.4	44.3	35.3	38.4
Agricultural area	36.5	29.8	45.8	40.3
Transitional woodland-shrub	16.3	23.8	18.1	16.4
Other	6.8	2.2	0.9	4.9

Supplementary Table S5: Results of pairwise Fisher Exact Tests of the distribution of landscape types for ASF-negative carcasses and ASF-positive carcasses found dead, ASF-negative hunted wild boar and random points. Since subsequent pairwise testing was performed, Bonferroni correction was applied. Therefore, p-values < 0.008 were considered significant and shown in bold.

	ASF-negative carcasses (n = 606)	ASF-positive carcasses (n = 1,444)	ASF-negative hunted wild boar (n = 9,527	Random points (n = 10,000)
ASF-negative carcasses		**< 0.001**	**< 0.001**	0.08
ASF-positive carcasses			**< 0.001**	**< 0.001**
ASF-negative hunted wild boar				**< 0.001**

Supplementary Table S6: Proportion of carcasses (in %) that were found or shot in forests, agricultural area, transitional areas and other areas (including waterbodies, wetlands, open spaces with little or no vegetation, urban areas and scrub and/or herbaceous vegetation associations) grouped by season. Records from April to September were considered as "summer" and records from October to March were considered as "winter".

	ASF-negative carcasses		ASF-positive carcasses		ASF-negative hunted	
	Summer (n = 317)	Winter (n = 289)	Summer (n = 826)	Winter (n = 618)	Summer (n = 3825)	Winter (n = 5,702)
Forest	40.7	40.1	42.6	46.4	29.5	39.2
Agricultural area	33.1	40.1	33.5	24.8	53.9	40.4
Transition	18.9	13.5	22.0	26.1	15.8	19.6
Other	7.3	6.2	1.8	2.8	0.7	0.9

Landscape composition

Supplementary Table S7: Median proportions (in %) of agricultural area, forest, transitional woodland-shrub, urban area, waterbodies and wetlands in a buffer zone of 3 km radius around ASF-negative carcasses, ASF-positive carcasses and ASF-negative hunted wild boar. Results are shown for animals found or hunted, respectively, in summer months (April to September) and in winter months (October to March). P-values show the results of Mann-Whitney-U testing of the summer group compared to the winter group. P-values < 0.05 are shown in bold.

	ASF-negative carcasses			ASF-positive carcasses			ASF-negative hunted wild boar		
	Summer (n = 317)	Winter (n = 289)	p	Summer (n = 826)	Winter (n = 618)	p	Summer (n = 3,825)	Winter (n = 5,702)	p
Agriculture	36.9	38.8	0.8	39.7	38.7	0.07	40.9	37.8	**< 0.001**
Forest	38.6	36.7	0.3	38.8	37.6	0.5	37.3	38.6	**< 0.001**
Woodland shrub	14.6	14.4	0.7	16.7	18.5	**0.004**	16.6	17.7	**< 0.001**
Urban	0.4	0.8	0.4	0.0	0.0	0.3	0.0	0.0	**0.002**
Water bodies	0.0	0.0	0.7	0.0	0.0	0.8	0.0	0.0	0.7
Wetlands	0.0	0.0	0.3	0.0	0.0	0.07	0.0	0.0	**< 0.001**

Distance measurements

Supplementary Table S8: Distances (m) of ASF-negative carcasses, ASF-positive carcasses and ASF-negative hunted wild boar to the next waterbodies, roads, settlements and forest edges grouped by season. Finding or shooting dates, respectively, from April to September were considered as "summer" and dates from October to March were considered as "winter". P-values show the results of Mann-Whitney-U testing of distance measurements from summer-group compared to winter-group. P-values < 0.05 are shown in bold.

	ASF-negative carcasses			ASF-positive carcasses			ASF-negative hunted wild boar		
	Summer	Winter	p	Summer	Winter	p	Summer	Winter	p
	(n = 317)	(n = 289)		(n = 826)	(n = 618)		(n = 3,825)	(n = 5,702)	
Waterbody	121	106	0.7	121	120	0.9	120	120	0.9
Road	816	402	**< 0.001**	1,152	1,226	0.3	1,145	1,282	**< 0.001**
Settlement	364	344	0.2	429	493	**0.005**	469	511	**< 0.001**
Forest edge	56	52	0.2	43	58	**0.001**	53	51	0.4

Generalized estimation equation model

Supplementary Table S9: Moran's I and Geary's C and p-values calculated on the standardized deviance residuals of ordinary univariable GLM models and based on 1,000 permutations.

Predictors	Moran's I	P-Value	Geary's C	P-Value
Distance to forest edge	0.3026	< 0.001	0.6767	< 0.001
Distance to road	0.2997	< 0.001	0.6758	< 0.001
Distance to waterbodies	0.3103	< 0.001	0.6687	< 0.001
Distance to settlement	0.3046	< 0.001	0.6723	< 0.001
Proportion of forest (total)	0.3102	< 0.001	0.6687	< 0.001
Proportion of coniferous forest	0.3001	< 0.001	0.6772	< 0.001
Proportion of broad-leaved forest	0.3003	< 0.001	0.6767	< 0.001
Proportion of mixed forest	0.3102	< 0.001	0.6687	< 0.001
Proportion of agricultural area	0.3068	< 0.001	0.6719	< 0.001
Proportion of transitional woodland-shrub	0.3016	< 0.001	0.6764	< 0.001
Proportion of urban area	0.2687	< 0.001	0.7074	< 0.001
Proportion of scrub	0.3101	< 0.001	0.6691	< 0.001
Proportion of open space	0.2938	< 0.001	0.6824	< 0.001
Proportion of wetlands	0.3096	< 0.001	0.6691	< 0.001
Proportion of waterbodies	0.2872	< 0.001	0.6887	< 0.001

Supplementary Table S10: Parameters (τ^2= nugget, σ^2 = sill, ϕ = range) of exponential models fitted to the semi-variograms calculated on the standardized deviance residuals of ordinary univariable GLM models and approximated practical range calculated according to Cressie (1993).

Predictors	τ^2	σ^2	ϕ	Practical Range
Distance to forest edge	0.569	0.626	10.576	31.68
Distance to road	0.556	0.616	10.859	32.53
Distance to waterbodies	0.566	0.631	10.613	31.79
Distance to settlement	0.575	0.609	10.429	31.24
Proportion of forest (total)	0.565	0.631	10.631	31.85
Proportion of coniferous forest	0.555	0.625	9.709	29.08
Proportion of broad-leaved forest	0.569	0.618	10.490	31.43
Proportion of mixed forest	0.565	0.631	10.631	31.85
Proportion of agricultural area	0.562	0.628	10.217	30.61
Proportion of transitional woodland-shrub	0.566	0.617	10.010	29.99
Proportion of urban area	0.553	0.588	9.032	27.06
Proportion of scrub	0.560	0.636	10.510	31.48
Proportion of open space	0.543	0.631	9.287	27.82
Proportion of wetlands	0.565	0.631	10.522	31.52
Proportion of waterbodies	0.560	0.608	9.535	28.56

Supplementary Table S11: Results of univariable GEE models showing estimates, p-values and the AUC of predictors. The outcome variable was the infection status of the carcasses found dead (ASF-negative [n = 605] versus ASF-positive [n = 1,423]) in Latvia for the time period from June 2014 to February 2021.

Predictors	Estimates	P-value	AUC
Distance to forest edge	-0.0345	0.048	0.530
Distance to road	0.0393	< 0.001	0.647
Distance to waterbodies	0.0009	0.472	0.501
Distance to settlement	0.0246	< 0.001	0.567
Proportion of forest (total)	0.0072	0.819	0.507
Proportion of coniferous forest	-0.0368	0.376	0.562
Proportion of broad-leaved forest	0.0210	0.744	0.581
Proportion of mixed forest	0.0072	0.819	0.507
Proportion of agricultural area	0.0150	0.574	0.532
Proportion of transitional woodland-shrub	0.1439	0.007	0.579
Proportion of urban area	-0.3443	0.002	0.622
Proportion of scrub	0.1345	0.800	0.502
Proportion of open space	-11.8531	0.002	0.525
Proportion of wetlands	0.3280	0.001	0.468
Proportion of waterbodies	-0.3217	< 0.001	0.556

Supplementary Figure S4: Semi-variogram and fitted exponential model of the multivariable GLM model that includes the predictors distance to the road, distance to forest edge, proportion of open space, proportion of waterbodies and proportion of mixed forest. The model parameters were estimated by weighted least squares. The parameter estimates were $\tau^2 = 0.541$, $\sigma^2 = 0.591$, $\phi = 9.172$ and the practical range (calculated according to Cressie (1993)) was 27.48 km.

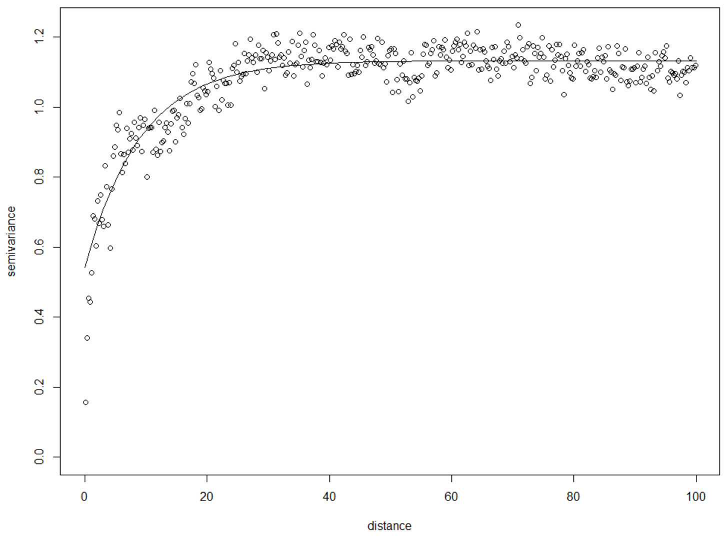

Manual analysis

Supplementary Table S12: Proportions of animals (in %) found or shot in forests, agricultural area, transitional woodland-shrub or in other landscape types according to manual categorization (based on orthophotos) and automated categorization (based on CLC map) for a smaller sample (n = 599). P-values show the results of the Fisher's Exact test of the distribution of locations of manual measurements compared to automated measurements (irrespective of the infection status of the carcasses).

	ASF-negative carcasses (n = 175)		ASF-positive carcasses (n = 249)		ASF-negative hunted wild boar (n = 175)		p-value
	Manual	Automated	Manual	Automated	Manual	Automated	
Forest	40.0	41.7	59.4	47.8	33.7	36.6	0.2
Agricultural area	31.4	38.3	17.7	20.5	43.4	46.9	0.1
Transitional woodland-shrub	14.9	12.6	20.1	29.3	21.7	16.6	0.5
Other	13.7	7.4	2.8	2.4	1.1	0.0	0.06
